# Needs Assessment for Interprofessional Education: Implications for Integration and Readiness for Practice

**DOI:** 10.3390/healthcare9040411

**Published:** 2021-04-02

**Authors:** Abdulraheem Almalki, Yoon Soo Park, Ara Tekian

**Affiliations:** 1 Department of Clinical Laboratory Sciences, College of Applied Medical Sciences, Taif University, P.O. Box 11099, Taif 21944, Saudi Arabia; 2 College of Medicine, University of Illinois at Chicago, Chicago, IL 60612, USA; yspark2@uic.edu (Y.S.P.); tekian@uic.edu (A.T.)

**Keywords:** interprofessional education, IPE, RIPLS, healthcare education, collaborative practice

## Abstract

Interprofessional education (IPE) is an important concept to promote health professionals for interprofessional collaboration. Successful implementation of IPE in health education programs requires consideration of readiness and effectiveness and faces some challenges/barriers. The aim of this study was to examine the perception, understanding and attitude of health profession students and faculty members toward IPE. A cross-sectional study was conducted with students and faculty members from six health professions at Taif University. The study involved administration of the Readiness for Inter-Professional Learning Scale (RIPLS) questionnaire to all students. In addition, focus groups were conducted separately with both students and faculty members. The study showed that only 10 participants (four students, six faculty members) indicated their previous knowledge of IPE. IPE remains a new approach for the majority of students and faculty members. There was no significant difference in the readiness of IPE between professions. Students and faculty members showed positive attitudes toward the IPE curriculum and they believe that it will improve medical education at our university.

## 1. Introduction

As described by the World Health Organization (WHO), interprofessional education (IPE) “occurs when students from two or more professions learn about, from, and with each other.” The goal is to prepare health profession students to work together and to provide safer health care in a collaborative practice-ready health workforce [1].

The “inter” in interprofessional requires the presence of all three prepositions “about, from, and with” in an interactive learning practice. IPE is meant to be part of the health profession education and not to replace education specific to each profession. Thus, each profession should maintain its own identity [2]. It should be integrated as a separate domain that includes four competencies: IP values/ethics, IP roles/responsibilities, IP communication and teamwork [3].

Currently interprofessional education is becoming a priority in global health care education programs. The purpose is to prepare health education students for interprofessional collaboration (IPC) for the future. IPC is claimed to have a positive impact on patient outcomes and the cost of health care [1]. Several accrediting bodies for health education programs have set standards for implementing IPE [4]. A framework of action has been developed by the WHO as a reference for health education programs. Specific IPE competencies were created by the Interprofessional Education Collaborative (IPEC) to maintain the quality of IPE programs (Interprofessional Education Collaborative, 2016). In addition, several assessment tools were created to help in the assessment of IPE program outcomes [5,6].

There are several examples of successfully implemented IPE models in the literature. Some models focus on shared basic coursework (centralized) and other rely on IPE activities outside the core course (decentralized). Both models have strengths and weaknesses.

For instance, the centralized model is easy to apply but will require administrative approval. The decentralized model, on the other hand, will not require administrative approval, in most cases, but will need further curricular revision.

The key factor for successful implementation of both models is having dedicated faculty members and a culture that support IPE [7].

At Taif University (TU), communication between health profession schools is minimal. Students from the different professions have no direct contact with each other. There are many issues related to IPE that we do not know. For example, what are students’ perceptions and understanding of IPE? What are faculty members’ understanding of IPE? Are there differences in student perceptions of IPE by profession? Are there differences in student and faculty members’ understanding of IPE? Are there differences in their attitude toward the IPE curriculum? Thus, students and faculty members need to be educated about IPE; their perception, understanding and attitude need to be measured and a plan of action to integrate IPE should be considered. This study aims to examine student and faculty perception about IPE. Moreover, this study also addresses student and faculty understanding of IPE and their attitude toward the IPE curriculum.

By integrating IPE as part of the curriculum for all students in the health professions, administrators would make students aware of their roles as health care providers within a team, and they would know how to communicate and collaborate with other members of the team. This consequently results in a safer healthcare practice.

## 2. Materials and Methods

### 2.1. Research Design

This study firstly aimed to explore students’ perceptions about IPE. Aa adapted version of the Readiness for Inter-Professional Learning Scale (RIPLS) original version, developed by McFadyen et al., 2005 [8], was administered to medical, pharmacy, laboratory sciences, nursing, radiology and physical therapy students (Supplementary Materials). Then, the students’ understanding of IPE and their attitude toward the IPE curriculum were investigated through focus groups. Six focus group sessions were carried out, one session for each profession. The proposed number of students in each group was 10. Similarly, faculty members’ (from all health professions) understanding of IPE and their attitude toward the IPE curriculum were investigated through focus groups as well. Six faculty members (three males and three females) were invited to each focus group session. In any department where there were enough Saudi and non-Saudi faculty members, two sessions were carried out. Questions of all focus groups were developed by the author and revised by supervisors.

### 2.2. Study Settings, Sample and Sampling Procedure

To measure student perception, all health profession students were invited to participate in the survey. Surveys were distributed from May to June 2017 as hard copies through the vice dean of research in each school (convenience sample). As informed, surveys were distributed to available students in all academic years at the time of data collection. Some academic years were not involved due to hospital rotations (in and out of Taif city): 6th year and 7th year medical students, 5th year pharmacy students and 4th year students in the college of applied medical sciences.

Following survey administration, focus groups were conducted from February to March 2018 with students, with one session for each profession. The average time for each session took around 1 h and 10 min. Participants were in their 3rd academic year (3rd of seven years for medicine, 3rd of six for pharmacy and 3rd of five years for other professions).

Third year students were selected due to their availability (most of their time was in their colleges, not hospitals) and because during the 3rd year the strength of their professional identity is not at the highest or lowest level.

For faculty members, approval was obtained from all professions except pharmacy. Approval of participation was not obtained from female medical faculty members and female non-Saudi nursing faculty members as well. Requests/invitations for participation were made several times but no responses were received. Seven focus groups were conducted from April to May 2018 as follows: two sessions with faculty members from clinical laboratory sciences, two sessions with faculty members from nursing, one session with faculty members from radiology, one session with faculty members from physical therapy and one session with faculty members from medicine. The average time for each session was one hour.

### 2.3. Data Collection

A previously published and validated questionnaire was used to measure student perception about IPE. The questionnaire has a 5-point rating scale: Strongly disagree, Disagree, Neutral, Agree and Strongly agree. For all items, Strongly disagree and Disagree were grouped together as negative response, and Agree and Strongly agree were grouped as a positive response. In addition, a focus group was utilized to investigate students’ and faculty members’ understanding of IPE, their attitude toward the IPE curriculum and to identify any differences in student perceptions of IPE by profession (Supplementary Materials). Notes were taking during the discussion, summarized at the end, and participants were asked for verification (member checking). Additionally, all focus groups were tape-recorded. Participants’ responses were then transcribed into a written text, grouped and categorized for analysis using a constant comparison analysis method. Questionnaires were administrated in both Arabic and English languages. Prior to administration, it was piloted on 15 health profession students. Students were asked what they thought about each item and what their response meant. All focus groups were conducted in Arabic language to ensure maximum input. Translation of the transcribed version (written text) was done as literally as possible by the author. It was then presented to two bilingual English–Arabic speakers, all of whom speak Arabic natively, to confirm accuracy (accuracy was 95%). The concepts being investigated in the questionnaire or in focus groups were easily transferred between cultures through direct translation (value-free). Before participation, approval to conduct the study was obtained from the vice dean for postgraduate studies and research at each health profession school. Informed consent from all participants was obtained. Institutional review board (IRB) approval was also obtained from Taif University Ethical Committee (Supplementary Materials).

### 2.4. Data Analysis

Microsoft Excel was used for data entry. Statistical Package for the Social Sciences (SPSS) version 21 was utilized for data analysis. Descriptive statistics were used to present the demographic data and present trends in data collected. For focus groups, responses were rearranged and grouped together for each question. Responses were then organized into categories and the main idea/theme, if any, was identified.

## 3. Results

### 3.1. Demographic

A total of 200 students from all health professions at Taif University (excluding radiology students) agreed to complete the questionnaire. Forty-eight questionnaires were returned incomplete or inappropriately completed (one answer for all questions, mainly neutral); they were excluded from the analysis.

The remaining 152 participants were: 49 medical students, 37 pharmacy students, 15 clinical laboratory sciences students, 25 nursing students and 24 physiotherapy students. Participants were from different academic years as shown in (Table 1).

### 3.2. Questionnaire Results

For the first nine items of the questionnaire (teamwork and collaboration), the majority of participants showed positive responses (agree) to all items (Table 2). Their responses may indicate that they valued cooperative learning and respected students from other health professions. The next seven items of the questionnaire were used to assess negative and positive professional identity.

The majority of students disagreed with all the items related to negative professional identity (items no. 10–12) and agreed with all items related to positive professional identity (items no. 13–16) (Table 2). Their responses were in agreement with the first part of the questionnaire (items no. 1–9) where they demonstrated their respect and the value of collaboration with other. The last part of the questionnaire (items no. 17–19) assessed participants’ perceptions about the roles and responsibilities of their own profession and others (Table 2).

The majority of participants (77.6%; 37 medicine students, 26 pharmacy students, 14 laboratory students, 23 nursery students and 18 physical therapy students) believed that the role of nurses and therapists is mainly to provide support for doctors. There were no differences between professions in their responses to this item, according to chi-squared test (*p* = 0.179).

For item no. 18, 41% of participants stated that they were not sure what their role will be. Based on chi-squared test (*p* < 0.001), there was a significant difference among professions and students’ years of study. None of the clinical laboratory sciences students and nursing students disagreed with this item (Table 3). For other professions, including medicine, pharmacy and physical therapy, the majority of participants selected the “Neutral” answer (medicine 21 of 51 students, pharmacy 12 of 37 students and physical therapy 11 of 24 students) (Table 3). Responses from the rest of participants were distributed between agreement and disagreement.

In addition, the majority of participants who agreed with this statement were 2nd and 3rd year students (Table 4). Finally, 123 (80.9%) of participants agreed that they have to acquire much more knowledge and skills than other health care students.

### 3.3. Focus Group Results

Students (including radiology students) and faculty members shared similar responses for most of the focus group questions (Table 5). The first key question was “Do you have direct contact with students/faculty members from other health professions?” The majority of both groups (46 students and 39 faculty members) indicated that they had direct contact with individuals from other professions, either socially or scientifically. Scientifically, for instance, students admitted that they discussed topics/cases informally with students from other professions. Faculty members stated that they taught students from other professions, they made and taught a shared subject; and they did collaborative research with others. Second, it seems that for the majority of students the roles and responsibilities of their own profession or of others were not yet clear. Three questions were used to explore understanding of roles and responsibilities: What do you think are their roles in the health care team? In what way do you think their roles are different from yours? In what way do you think their roles and your roles overlap? Student responses for these questions can be categorized into: students who did not know their roles or the roles of others (18 students), students who imprecisely knew their roles but not the roles of others (19 students) and students who knew imprecisely their roles and the roles of other (20 students). Although the roles and responsibilities of their own profession were clear for faculty members, the roles and responsibilities of others were not. Third, both groups were asked if they thought it was important to know the roles and responsibilities of other professions, and why did they think it was important. They believed that it was important that they knew the roles and responsibilities of others. They believed that knowing the roles and responsibilities of others would increase respect and lead to better teamwork, easy communication and fewer medical errors. Next, both groups were asked if they thought there was a shared area in the curriculum of health professions, and if they supported teaching together. Participants thought that there was a shared area between the curriculums of each profession and the majority of them supported teaching those shared areas together. Shared areas according to participants could be categorized into: basic sciences such as biology, general subjects such as medical ethics, and topics within certain clinical modules such as anemia. Basic sciences and general subjects would be normally found early in the curriculum while shared topics were scattered throughout the curriculum. There was an agreement among all participants that learning together, if implemented, should be done through practical sessions and interactive teaching strategies including: seminars, team-based learning (TBL), problem-based learning (PBL), clinical simulation and case studies. Finally, faculty members were asked if there were any considerations or limitations that we should think about before implementation. Faculty members believed that the available facilities/resources and resistance from faculty members/leaders would both need to be considered carefully ahead of implementation.

## 4. Discussion

Although IPE is considered as an important pedagogical approach for preparing health profession students to work in a collaborative environment, the majority of health profession students and faculty members at Taif University had not even heard of IPE before. This was clearly indicated in their responses to the questionnaire or in the focus groups.

Students seem to value collaborative learning and they seem to hold respect for students from other professions. They demonstrated positive responses (agreement) for the first nine items in the RIPLS questionnaire that measured readiness for teamwork and collaboration; concurring with several other studies [9,10,11,12,13]. This value of collaboration may be due to the fact that health profession students used to study preparatory year together and because they clearly stated that in the focus groups they are uncomfortable with not knowing the role of others. Students’ respect of the other was also indicated in their responses to items 10–16 in the RIPLS questionnaire (negative and positive professional identity). These findings were consistent with students’ responses from King Saud and King Abdulaziz Universities [14,15]. They were also consistent with students’ perceptions from different countries such as Iran and Australia [16,17].

Students’ responses to the questionnaire and focus group questions indicated their lack of knowledge (entirely or partially) about the roles and responsibilities of their own or others. These findings were in a disagreement with the results reported by Al-Eisa et al. (2016) and Dargahi et al. (2012) [14,16]. This may be because the majority of participants in Al-Eisa et al.’s (2016) [14] study and all participants in Dargahi et al. (2012) [16] were 4th year students, whereas the majority of our students were 2nd (55 students) and 3rd year students (56 students). In addition, the majority of students (from all professions) agreed that they have to acquire much more knowledge and skills than other health care students. This may emphasize the previous finding, which is that students have a poor understanding of their own roles. This finding was in agreement with student perceptions at King Saud University [14]. Similar to students, it seems that the exact role and responsibilities of each profession were not clear to faculty members. Only a limited number of faculty members managed to recall limited experiences that may have helped them in knowing the roles and responsibilities of others. Even when they became professional, they said there was always a job description for each profession that they had to follow precisely, thus knowing the roles of others was not a necessity.

Differences in the readiness of students for IPE between professions were reported in studies from America, Canada, New Zealand and Sweden [13,18,19]. However, for all questionnaire subscales/domains, there was no significant difference between professions or students’ years of study in this study. A similar finding was also reported in studies from the United Kingdom, the United States and Iran [20,21,22].

Student knowledge about the roles and responsibilities has been shown to improve after IPE interventions in several studies, in addition to improvements in student knowledge (objective knowledge), skills and attitude. Cohen et al. (2016) [23] used pre-post mixed methods in a protocol-driven training program with trainees from different health professions and measured their knowledge about Parkinson disease, team-based care, the role of other disciplines and attitudes towards health care teams. The result showed significant post-test improvement in all outcomes compared to the control. Similarly, Eccott et al. (2012) [24] designed, implemented and evaluated an IPE problem-based learning module in a Canadian university with a convenience sample of 24 students from different health professions. The results showed improvement in: student attitude toward IPE, student knowledge about the roles and student confidence to collaborate.

Prior to IPE implementation, future stakeholders should carefully consider the resources and resistance. Scheduling IPE activities with all the resources needed, including space and faculty members, may represent a real challenge. It will require class synchronization for all health professions to do shared activities. This is not easy, taking into consideration that each profession has its own curriculum and timetable. In addition, the college of applied medical science is not in close proximity to the pharmacy and medicine colleges. Scheduling on its own has been reported to be one of the main barriers for IPE [25,26]. Thus, careful planning ahead with administrators/leaders’ support is needed. Administrators first need to recognize the importance of IPE and the expected outcomes in order to direct resources to the newly required change (administrative-level resistance). If administrators’ support is obtained, faculty members will also need to recognize and value IPE to ensure effective operation. They may resist to avoid any extra load, especially since there is a shortage of faculty members in most professions. According to Hall and Zierler (2015) [27], a faculty development program to prepare IPE leaders can be a facilitator. The authors proposed a guide where eight academic institutions partnered to launch the program. The program received positive feedback from the participants and showed that it could create an effective community among participants.

IPE should be implemented early in the curriculum and gradually. Why early? Faculty members believe that it will be easier since the content of the 1st year and the 1st semester of the 2nd year are mostly shared across professions and because it will orient students to the collaborative environment from the beginning. Starting early in the curriculum is an important point and supported by other studies. For example, Coster et al. (2008) [21] found that readiness for IPE among health profession students was high on entry to university and gradually decreased afterwards. Gradual implementation would also reduce resistance until everyone starts valuing IPE. Additionally, it will help in revealing hidden challenges early and give room for re-planning and improvements.

## 5. Conclusions

Although IPE is mandatory in many health professional programs, it remains a new concept at Taif University, and health professional programs function separately. The presented study was aimed mainly to measure the perception, understanding and attitude of health profession students and faculty members toward IPE. Their perception and understanding were limited to a few examples mentioned by only a minority of participants. However, they showed a positive attitude toward IPE curriculum, and they believed it would improve medical education at our university.

Their attitude, if associated with dedication, will be the keystone for IPE initiation. Initiation of IPE, like any new idea, may face challenges and rejection. However, careful preparation and a high degree of organization, with stakeholders’ support, will make it a reality.

## Figures and Tables

**Table 1:** Student distribution per profession and academic year.

Profession	2nd Year	3rd Year	4th Year	5th Year	Total
Medicine	10	14	14	11	49
Pharmacy	9	14	14		37
Clinical laboratory sciences	10	5	-	-	15
Nursing	12	13	-	-	25
Physiotherapy	14	10	-	-	24
Total	55	56	28	13	152

**Table 2:** Students’ readiness for interprofessional education using the Readiness for Inter-Professional Learning Scale (RIPLS).

Domain	No.	Item	Agree
Teamwork and collaboration	1	Learning with other students will help me become a more effective member of a health care team	119 (78.3%)
	2	Patients would ultimately benefit if health care students worked together to solve patient problems	128 (84.2%)
	3	Shared learning with other health care students will increase my ability to understand clinical problems	123 (78.9%)
	4	Learning with health care students before qualification would improve relationships after qualification	120 (78.9%)
	5	Communication skills should be learned with other health care students	115 (75.6%)
	6	Shared learning will help me to think positively about other professionals	125 (82.2%)
	7	For small groups learning to work, students need to trust and respect each other	135 (88.8%)
	8	Team-working skills are essential for all health care students to learn	135 (88.8%)
	9	Shared learning will help me to understand my own limitations	109 (71.7%)
Negative professional identity	10	I don’t want to waste my time learning with other health care students	29 (20%)
	11	It is not necessary for undergraduate health care students to learn together	26 (17.1%)
	12	Clinical problem-solving skills can only be learned with students from my own department	33 (21.7%)
Positive professional identity	13	Shared learning with other health care students will help me to communicate better with patients and other professionals	98 (64.5%)
	14	I would welcome the opportunity to work on small group projects with other health care students	118 (77.6%)
	15	Shared learning will help to clarify the nature of patient problems	129 (84.9%)
	16	Shared learning before qualification will help me become a better team worker	135 (88.8%)
Roles and responsibilities	17	The function of nurses and therapists is mainly to provide support for doctors	118 (77.6%)
	18	I’m not sure what my professional role will be	63 (41.4%)
	19	I have to acquire much more knowledge and skills than other health care students	123 (80.9%)

**Table 3:** Distribution of student responses to item no. 18 of RIPLS questionnaire per profession.

	Clinical Laboratory Sciences	Nursing	Medicine	Pharmacy	Physical Therapy
Strongly agree	7	8	4	3	1
Agree	6	13	7	8	6
Neutral	2	4	21	12	11
Disagree	0	0	13	7	6
Strongly disagree	0	0	6	7	6
Total	15	25	51	37	24

**Table 4:** Distribution of student responses to item no. 18 per academic year of RIPLS questionnaire.

	2nd Year	3rd Year	4th Year	5th Year
Strongly agree	16	7	0	0
Agree	13	23	4	0
Neutral	15	14	14	7
Disagree	9	8	5	4
Strongly disagree	2	4	5	2
Total	55	56	28	13

**Table 5:** Summary of responses by students and faculty members to focus group questions.

Item	Perceptions	
	Students (Total No. 57)	Faculty (Total No. 44)
Direct contact with individuals from other professions	Yes (46)	Yes (39)
Roles and responsibilities of own profession	Not precisely clear (57)	Clear (44)
Roles and responsibilities of other professions	Not precisely clear (57)	Not precisely clear (44)
Is it important to know the roles and responsibilities of other profession?	Yes (34)	Yes (44)
Is there shared content within the curriculum of each profession?	Yes (57)	Yes (44)
Supporting studying together	Yes (57)	Yes (31)
Favorable teaching methods	Practical, case study, TBL, PBL, simulation	Practical, case study, TBL, PBL, simulation

## Supplementary Materials

The following are available online at https://www.mdpi.com/article/10.3390/healthcare9040411/s1, RIPLS questionnaire. Student focus group questions. Faculty members focus group questions. IRB approval.
