# Supplementary material for: Needs Assessment for Interprofessional Education: Implications for Integration and Readiness for Practice
Source: Healthcare (Basel). 2021 Apr 2;9(4):411. doi: 10.3390/healthcare9040411 (PMC8066906; doi:10.3390/healthcare9040411)
Supplement: Supplementary file 1 [file healthcare-09-00411-s001.zip › Supplementary files/Faculty focus group.pdf]

FOCUS GROUP

Introduction

Welcome
Thanks for agreeing to be part of the focus group. I appreciate your willingness to participate.

Purpose of focus groups
The reason we are having these focus groups is to find out your understanding of IPE and how it might change after an educational intervention.
I need your input and want you to share your honest and open thoughts with me.

Ground rules
1. The focus group will last for approximately 60-90 minutes

2. I want you to do the talking.
We would like everyone to participate.
I may call on you if I haven't heard from you in a while.

3. There are no right or wrong answers
Every person's experiences and opinions are important. Speak up whether you agree or disagree.
I want to hear a wide range of opinions.

4. What is said in this room stays here
I want folks to feel comfortable sharing when sensitive issues come up.

5. I will be tape recording the group
I want to capture everything you have to say.
I don't identify anyone by name in our report. You will remain anonymous.

Opening questions

Have you heard of the IPE?

What is your perceptions/ understanding of IPE?

Have you seen any role model before?

Key questions

Question	Probing question
Do you have a direct contact with faculty/students from other health profession?	Do you perform activities together (teaching/research/ patient care)?
In which way do you think the roles of health professions are different/ overlap?	
Do you think your students know their roles and the roles of others?	
Do you think it is important for health profession students to understand each other's roles and responsibilities?	Why do think that?
Do you think there is a shared area between the curriculums of each profession?	Can you give example?
Should we teach students from different professions those shared area together?	Why?<br>If yes, How?
In addition to those shared area, what else can we do to implement IPE successfully?	
Are there any considerations/ limitations we should think about?	How to overcome?
Do you think IPE will have an impact on student's future career/ patient care?	If yes, How?

Summing up
By the moderator

Closing question
Is there anything that you think is important/related to the context and has not been brought up in the discussion?
