# Supplementary material for: Needs Assessment for Interprofessional Education: Implications for Integration and Readiness for Practice
Source: Healthcare (Basel). 2021 Apr 2;9(4):411. doi: 10.3390/healthcare9040411 (PMC8066906; doi:10.3390/healthcare9040411)
Supplement: Supplementary file 1 [file healthcare-09-00411-s001.zip › Supplementary files/RIPLS.pdf]

Readiness for Interprofessional Learning Scale (RIPLS) Questionnaire

-

The purpose of this questionnaire is to examine the health professions students' perceptions of interprofessional education.

الهدف من هذه الدراسة هو معرفة تصور طلاب التخصصات الصحية عن التعليم التداخلي /المشترك

Interprofessional Education "occurs when students from two or more professions learn about, from, and with each other" (Baker, 2010). For instance, a group of students from different health professions (medicine, pharmacy, laboratory science, nursing and physical therapy) are brought together as a team in a classroom/clinic to discuss their roles in a given clinical scenario.
IPE is meant to be part of health profession education and not to replace education specific to each profession. Thus, each profession should maintain its own identity

التعليم التداخلي/ المشترك يحدث عندما يتعلم طلاب التخصصات الصحية المختلفة من بعضهم،مع بعضهم عن موضوع طبي مشترك لكل منهم دوره الخاص فيه. على سبيل المثال: عندما يكون هناك فريق عمل مكون من طلاب كلية الطب، طلاب كلية الصيدلة و طلاب كلية العلوم
االطبية في قاعة دراسية أو مستشفى لمناقشة حاله سريريه ويقدم كلا منهم رأيه بما يتناسب مع تخصصه.
التعلم التداخلي لا يعني دمج جميع التخصصات الصحية في تخصص واحد بل يعني أن يحافظ كل تخصص على هويته الخاصه ومنهجه مع الحرص على أن يخصص جزء من الخطة الدراسية لاتاحة الفرصة لطلاب التخصصات الصحية المختلفة للتعلم مع بعضهم في بيئة تعليمية مشتركة

I agree to participate in this study / أوافق على المشاركة

Yes/ نعم	No/ لا
○	○

Your profession/ التخصص

Medicine/ طب	Pharmacy/ صيدلة	Medical laboratory / مختبرات طبية	Nursery/ تمريض	Physical therapy/ علاج طبيعي
○	○	○	○	○

Have you had any previous experience of interprofessional education/ هل كان لك تجربة سابقة في التعليم التداخلي

Yes/ نعم	No/ لا
○	○

- If you answered **yes**, please give a brief statement about your previous experience and its impact

اذا كان الجواب "نعم" الرجاء تقديم وصف مختصر لتجربتك السابقة

Learning with other students will help me become a more effective member of a health care team

التعلم مع طلاب التخصصات الصحية الأخرى سيساعدني على أن أكون عضو أكثر فعالية في الفريق الطبي

Strongly agree/ موافق بشدة	Agree/ موافق	Neutral / محايد	Disagree/ غير موافق	Strongly disagree/ غير موافق بشدة
○	○	○	○	○

Patients would ultimately benefit if health-care students worked together to solve patient problems

سيكون هناك فائدة حتمية للمرضى اذا عمل طلاب التخصصات الصحية المختلفة جنبا الى جنب لحل مشاكل المرضى

Strongly agree/ موافق بشدة	Agree/ موافق	Neutral/ محايد	Disagree/ غير موافق	Strongly disagree/ غير موافق بشدة
○	○	○	○	○

Shared learning with other health-care students will increase my ability to understand clinical problems

التعلم المشترك مع طلاب التخصصات الصحية الأخرى سيزيد من قدرتي على فهم مشاكل المرضى السريرية

Strongly agree/ موافق بشدة	Agree/ موافق	Neutral/ محايد	Disagree/ غير موافق	Strongly disagree/ غير موافق بشدة
○	○	○	○	○

Learning with health-care students before qualification would improve relationships after qualification

التعلم التداخلي مع طلاب التخصصات الصحية الأخرى قبل التخرج سيساعد في تطوير التعاون والعلاقات بين أعضاء الفريق الطبي بعد التخرج

Strongly agree/ موافق بشدة	Agree/ موافق	Neutral/ محايد	Disagree/ غير موافق	Strongly disagree/ غير موافق بشدة
○	○	○	○	○

Communication skills should be learned with other health- care students

يفضل أن يكون تعلم مهارات التواصل بشكل جماعي مع طلاب التخصصات الصحية الأخرى

Strongly agree/ موافق بشدة	Agree/ موافق	Neutral/ محايد	Disagree/ غير موافق	Strongly disagree/ غير موافق بشدة
○	○	○	○	○

Shared learning will help me to think positively about other professionals

التعلم المشترك مع طلاب التخصصات الصحية الأخرى سيساعدني على أن أنظر لتخصصاتهم نظرة ايجابية

Strongly agree/ موافق بشدة	Agree/ موافق	Neutral/ محايد	Disagree/ غير موافق	Strongly disagree/ غير موافق بشدة
○	○	○	○	○

For small group learning to work, students need to trust and respect each other

لنجاح حلقات النقاش المصغرة، يحتاج طلاب التخصصات الصحية المختلفة إلى الثقة والاحترام المتبادل لبعضهم البعض

Strongly agree/ موافق بشدة	Agree/ موافق	Neutral/ محايد	Disagree/ غير موافق	Strongly disagree/ غير موافق بشدة
○	○	○	○	○

Team-working skills are essential for all health care students to learn

تعلم مهارات العمل الجماعي مهم لجميع طلاب التخصصات الصحية

Strongly agree/ موافق بشدة	Agree/ موافق	Neutral/ محايد	Disagree/ غير موافق	Strongly disagree/ غير موافق بشدة
○	○	○	○	○

Shared learning will help me to understand my own limitations

التعلم المشترك سيساعدني على فهم ومعرفة حدودي و امكانياتي

Strongly agree/ موافق بشدة	Agree/ موافق	Neutral/ محايد	Disagree/ غير موافق	Strongly disagree/ غير موافق بشدة
○	○	○	○	○

I don’t want to waste my time learning with other health care students

أنا لا أرغب في تضييع واستنزاف وقتي في التعلم مع طلاب التخصصات الأخرى

Strongly agree/ موافق بشدة	Agree/ موافق	Neutral/ محايد	Disagree/ غير موافق	Strongly disagree/ غير موافق بشدة
○	○	○	○	○

It is not necessary for undergraduate health-care students to learn together

ليس من الضروري لطلاب التخصصات الصحية في مرحلة البكالوريوس التعلم بشكل تداخلي مع بعضهم البعض

Strongly agree/ موافق بشدة	Agree/ موافق	Neutral/ محايد	Disagree/ غير موافق	Strongly disagree/ غير موافق بشدة
○	○	○	○	○

Clinical problem-solving skills can only be learned with students from my own department

يمكن تعلم مهارات حل المشاكل السريرية فقط مع طلاب من ذات التخصص/ الكلية

Strongly agree/ موافق بشدة	Agree/ موافق	Neutral/ محايد	Disagree/ غير موافق	Strongly disagree/ غير موافق بشدة
○	○	○	○	○

Shared learning with other health-care students will help me to communicate better with patients

التعلم المشترك مع طلاب التخصصات الصحية الأخرى سيساعدني في التواصل بشكل أفضل مع المرضى

Strongly agree/ موافق بشدة	Agree/ موافق	Neutral/ محايد	Disagree/ غير موافق	Strongly disagree/ غير موافق بشدة
○	○	○	○	○

I would welcome the opportunity to work on small-group projects with other health-care students

أرحب بأي فرصة للعمل في مشروع صغير مع طلاب من تخصصات صحية مختلفة

Strongly agree/ موافق بشدة	Agree/ موافق	Neutral/ محايد	Disagree/ غير موافق	Strongly disagree/ غير موافق بشدة
○	○	○	○	○

Shared learning will help to clarify the nature of patient problems

التعلم المشترك سيساعد في فهم طبيعية مشاكل المرضى الصحية

Strongly agree/ موافق بشدة | Agree/ موافق | Neutral/ محايد | Disagree/ غير موافق | Strongly disagree/ غير موافق بشدة

Shared learning before qualification will help me become a better team worker

التعلم المشترك قبل التخرج سيساعدني لأن أكون عضو أفضل في الفريق الطبي مستقبلا

Strongly agree/ موافق بشدة | Agree/ موافق | Neutral/ محايد | Disagree/ غير موافق | Strongly disagree/ غير موافق بشدة

The function of nurses and therapists is mainly to provide support for doctors

وظيفة الممرضين و غيرهم من الاخصائين بشكل أساسي تكمن في تقديم الدعم للأطباء

Strongly agree/ موافق بشدة | Agree/ موافق | Neutral/ محايد | Disagree/ غير موافق | Strongly disagree/ غير موافق بشدة

I'm not sure what my professional role will be

لست متأكد بعد ماذا سيكون دوري في الفريق الطبي مستقبلا

Strongly agree/ موافق بشدة | Agree/ موافق | Neutral/ محايد | Disagree/ غير موافق | Strongly disagree/ غير موافق بشدة

I have to acquire much more knowledge and skills than other health-care students

يجب علي أن اكتسب معرفة ومهارات أكثر من طلاب التخصصات الصحية الأخرى

Strongly agree/ موافق بشدة | Agree/ موافق | Neutral/ محايد | Disagree/ غير موافق | Strongly disagree/ غير موافق

Please, add any comments or recommendations you find relevant

الرجاء، إضافة أي تعليق أو توصية تعتقد أنها ذات علاقة بالتعليم التداخلي
