# Supplementary material for: Needs Assessment for Interprofessional Education: Implications for Integration and Readiness for Practice
Source: Healthcare (Basel). 2021 Apr 2;9(4):411. doi: 10.3390/healthcare9040411 (PMC8066906; doi:10.3390/healthcare9040411)
Supplement: Supplementary file 1 [file IRB.pdf › Supplementary files/IRB.pdf]

Kingdom of Saudi Arabia
Ministry of Higher Education
Taif University
Research Ethics Committee

المملكة العربية السعودية
وزارة التعليم العالي
جامعة الطائف
لجنة أخلاقيات البحث العلمي

جامعة الطائف
TAIF UNIVERSITY

Committee Decision

Dear Dr. Abdulraheem Ali Almalki

Application No.: 38-36-0033

On behave of the membership of the research ethics committee, we would like to inform you that a decision has been made with regards to your research proposal entitled: **Need Assessment and Educational Intervention for Interprofessional Education: Implications for Integration and Readiness for Interprofessional Practice.**

The decision on your research proposal which is expected to be executed during the between **May 2017 to May 2018 is:**

- [√] **Approved**
- [ ] **Approved after amendments (please see enclosed file)**
- [ ] **Not approved (please see enclosed file)**

Kind regards,

Dr. Mohammed Yaslam Shobrak

Chairman of the committee
Date:

رقم القيد : ٦١٣٥٠/عام
تاريخ الإحالة : ٠٣ / ٠٩ / ١٤٣٨ هـ
المرفقات : لا يوجد
WWW.TU.EDU.SA

جامعة الطائف - المجلس العلمي
أمين المجلس
Secretary General
Taif University - Academic Council

Scanned with CamScanner
